# Investigation of fatal human Borna disease virus 1 encephalitis outside the previously known area for human cases, Brandenburg, Germany – a case report

**DOI:** 10.1186/s12879-021-06439-3

**Published:** 2021-08-10

**Authors:** Dennis Tappe, Kirsten Pörtner, Christina Frank, Hendrik Wilking, Arnt Ebinger, Christiane Herden, Christoph Schulze, Birgit Muntau, Petra Eggert, Petra Allartz, Gerlind Schuldt, Jonas Schmidt-Chanasit, Martin Beer, Dennis Rubbenstroth

**Affiliations:** 1 Bernhard Nocht Institute for Tropical Medicine, Bernhard-Nocht-Str. 74, 20359 Hamburg, Germany; 2 Department for Infectious Disease Epidemiology, Robert Koch Institute, Berlin, Germany; 3 Postgraduate Training for Applied Epidemiology (PAE) affiliated with the European Programme for Intervention Epidemiology Training (EPIET), European Centre for Disease Prevention and Control (ECDC), Stockholm, Sweden; 4 Institute of Diagnostic Virology, Friedrich-Loeffler-Institut, Greifswald-Riems, Germany; 5 Institute for Veterinary Pathology, Justus-Liebig-University Gießen, Gießen, Germany; 6 Landeslabor Berlin-Brandenburg, Frankfurt (Oder), Germany

**Keywords:** Bornavirus, BoDV-1, Clinical awareness, Phylogeny, Case report

## Abstract

**Background:**

The true burden and geographical distribution of human Borna disease virus 1 (BoDV-1) encephalitis is unknown. All detected cases so far have been recorded in Bavaria, southern Germany.

**Case presentation:**

A retrospective laboratory and epidemiological investigation of a 2017 case of fatal encephalitis in a farmer in Brandenburg, northeast Germany, demonstrated BoDV-1 as causative agent by polymerase chain reaction, immunohistochemistry and in situ hybridization. Next-generation sequencing showed that the virus belonged to a cluster not known to be endemic in Brandenburg. The investigation was triggered by a recent outbreak of animal Borna disease in the region. Multiple possible exposures were identified. The next-of-kin were seronegative.

**Conclusions:**

The investigation highlights clinical awareness for human BoDV-1 encephalitis which should be extended to all areas endemic for animal Borna disease. All previously diagnosed human cases had occurred > 350 km further south. Further testing of shrews and livestock with Borna disease may show whether this BoDV-1 cluster is additionally endemic in the northwest of Brandenburg.

## Background

Borna disease virus 1 (BoDV-1) is a zoonotic virus of the *Bornaviridae* family that is harbored by the bicolored white-toothed shrew (*Crocidura leucodon*) as natural reservoir. The virus has long been known for causing animal Borna disease (BD), a non-purulent meningomyelo-encephalitis of mainly horses and sheep in endemic regions of Germany, Liechtenstein, Switzerland and Austria [1]. Since 2018, the zoonotic potential of BoDV-1 has been demonstrated by at least 17 naturally acquired sporadic or transplant-related human encephalitis cases with 16 fatalities [2–7]. All sporadic human BoDV-1 cases occurred in the federal state of Bavaria in Southern Germany. BoDV-1 is related to, but distinct from, the variegated squirrel bornavirus 1 (VSBV-1) [8].

Here, we report investigations for a BoDV-1 etiology of a 2017 case of fatal human encephalitis with unknown cause in the federal state of Brandenburg, Germany. The likely place of infection lies on the northern fringe of the geographical area known to be endemic for BD on the North German plains. In March 2020, BoDV-1 infections were reported from an alpaca farm and two horses within a 35 km radius surrounding the patient’s home [9].

## Case presentation

### Clinical disease

A 59-year-old German farmer from the northwest of the federal state of Brandenburg developed headache, malaise and fatigue in early March 2017. No relevant medical preconditions were known. Within days, he experienced nausea, psychomotor slowing, apathy and temporary sensory aphasia. He was admitted to a local hospital with ataxia and dyspnea a few days later. Cranial magnetic resonance imaging (MRI) and lumbar puncture (LP) were normal on admission. After developing high fever, paraplegia and coma, he was transferred to intensive care unit for invasive ventilation. Due to pneumogenic sepsis, the patient received broad-spectrum antibiotics, antimycotics, and aciclovir. Broad-range pathogen testing from blood and cerebrospinal fluid (CSF) was negative, and repeat MRI about 3 weeks post onset remained normal. However, repeat LP a few days later showed lympho-monocytic pleocytosis (109 cells/μl; normal < 4), with marked CSF protein elevation (1951 mg/L; normal < 450) and lactate increase (5.49 mmol/L; normal < 2.4). Assuming paraneoplastic limbic encephalitis based on positive CRMP5/CV2 and Hu (ANNA-1) serum autoantibodies, the patient received additional high dose corticosteroid treatment. Four weeks after symptom onset (3 weeks after hospitalization), brain death was diagnosed and life support was stopped. Brain histopathological examination revealed necrosis and non-purulent inflammation with microglia expansion and perivascular lymphocyte cuffing, consisting mainly of T lymphocytes. Meninges also showed lymphocyte infiltration. The tentative diagnosis was fatal meningoencephalitis of unknown etiology, likely viral or paraneoplastic.

### Retrospective laboratory analyses

**Fig. 1:**
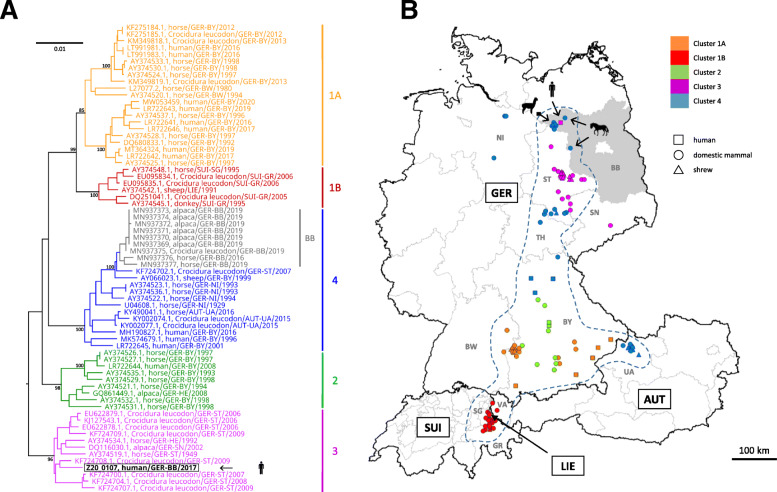
Phylogeny and geography of BoDV-1 from endemic areas. **A** Phylogenetic analysis of BoDV-1 sequences. Representative partial human and animal BoDV-1 sequences (1824 nucleotides, representing genome positions 54 to 1877 of BoDV-1 reference genome U04608) from the endemic regions in Germany (GER), Austria (AUT), Switzerland (SUI) and Liechtenstein (LIE) were analysed by Neighbor-Joining algorithm and Jukes-Cantor distance model in Geneious Prime. The tree was rooted using sequence BoDV-2 No/98 (AJ311524, not shown). The sequence generated in this study is depicted in black and bold. Previously published sequences from alpacas and horses from the federal state of Brandenburg (BB) are depicted in grey. Values at branches represent support in 1000 bootstrap replicates. Only bootstrap values ≥70 at major branches are shown. Cluster designations, host and geographic origin are indicated according to previously published work [1–3, 7, 9–13]. **B** The human case reported in this study is presented together with previously published BoDV-1 infections of alpacas and horses in Brandenburg and sequence-confirmed BoDV-1 infections of shrews (triangles), domestic mammals (circles) and humans (squares) with available geographic localization in known endemic regions [1–3, 7, 9–13]. Colors represent regional BoDV-1 sequence clusters (see Figure 1A). Human cases are generally marked at the centre of their county of residence. Imprecise geographic information and/or aberrant infection sources due to travel cannot be excluded for humans and domestic mammals. The area within the dashed blue line represents the presumed endemic area of BoDV-1. Grey letters represent federal states in Germany (BB: Brandenburg; BW: Baden-Wuerttemberg; BY: Bavaria; HE: Hesse; NI: Lower Saxony; SN: Saxony; ST: Saxony-Anhalt; TH: Thuringia) and Austria (UA: Upper Austria; VA: Vorarlberg) and cantons in Switzerland (GR: Grisons; SG: St. Gall) for which confirmed BoDV-1 infections are presented

**Fig. 2:**
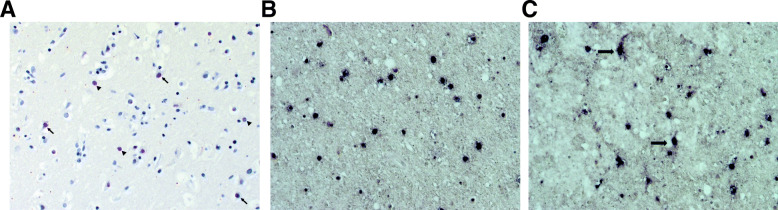
Demonstration of BoDV-1 antigen, BoDV-1 genomic RNA and viral mRNA. **A** BoDV-1 phosphoprotein (P) is demonstrated as intranuclear inclusions of neurons (arrows) and astrocytes (arrowheads) in midbrain. Diaminobenzidine immunostain with hematoxylin counterstain with 200-fold magnification. **B** Genomic BoDV-1 RNA (N gene) is present only in nuclei of brain cells. Midbrain, 100-fold magnification. **C** The respective viral mRNA (N gene) is also predominantly located in the nuclei of brain cells, with few cells also displaying cytoplasmic signals (arrows). Midbrain, 100-fold magnification

A possible BoDV-1 etiology of this cryptic encephalitis case was retrospectively investigated 3 years post mortem, triggered by media reports on zoonotic BoDV-1 infections [2] and by recent BoDV-1 infections reported from alpacas at a farm near to the patient’s home [9]. Neither serum nor CSF of the patient were available for serological examination. However, archived formalin-fixed paraffin-embedded (FFPE) brain and peripheral organ samples of the patient were provided by the pathologists who had conducted the autopsy in 2017. BoDV-1-specific real-time reverse transcription polymerase chain reactions (RT-qPCRs) [2, 3] from all FFPE brain specimens (various cerebrocortical regions, basal ganglia, hippocampus, cerebellum and brainstem) were positive after RNA extraction using the miRNeasy FFPE Kit (Qiagen, Hilden, Germany), with cycle-of-threshold (ct) values of 16.9–22.6 (equaling ~ 10^7^ copies/μg extracted RNA). These ct values are rather low as compared to FFPE material from previously detected human cases [2], which may be a result of the true viral load as well as of the RNA quality of the sample. Peripheral organs (kidneys, heart, lungs, spleen and liver) were negative. Phylogenetic analysis of the BoDV-1 sequence (GenBank accession number MT515369) assembled by high throughput-sequencing [2] grouped the virus into regional BoDV-1 cluster 3 (Fig. 1A). Cluster 3 is known to be endemic in parts of the neighboring federal states of Saxony-Anhalt and Saxony, whereas known BoDV-1 sequences from alpacas and horses in Brandenburg so far belonged to cluster 4 [9] (Fig. 1A and B). Immunohistological examination for BoDV-1 P protein [14] in FFPE brain samples showed positive immunostaining of neurons and astrocytes with intranuclear inclusions (Fig. 2A). In situ hybridization [3, 15] showed predominantly nuclear signals for viral genomic RNA and mRNA, with only few cells displaying also cytoplasmic viral mRNA signals (Fig. 2B and C). The final diagnosis of a fatal BoDV-1 encephalitis was established 3 years after the farmer’s death, in January 2020.

### Epidemiological investigations

The patient’s next-of-kin were interviewed about the farm and possible exposures of the patient in detail. The patient’s farm, consisting of one residential building and three barns, is located in the northwest of Brandenburg (Fig. 1B) on the margins of a hamlet. Small numbers of horses, pigs, geese, cattle, goats, ducks, rabbits and chicken had been kept on the farm until 2015. The farmer had contact to the neighbor’s dog and had kept seven outdoor cats. The cats regularly brought dead small mammals to the residential building. Raccoons and hedgehogs were attracted by left-out cat food; unknown animals accessed trash bags stored in a barn. At the time of the farmer’s death, approximately 100 sheep were kept in one barn and on adjacent fields. Hay for the sheep was bought from a neighboring farm and stored in the barn. From August 2016 through April 2017, 10 sheep had died from a neurological disorder of unknown etiology. FFPE brain samples from two lambs and one ewe that had died a few days after the farmer’s death were available for retrospective analysis by BoDV-1-specific RT-qPCR and yielded negative results.

The farmer did not leave the region for years. His routine farm work consisted of caring for the animals, buildings and grounds, but not gardening. Dead animals were handled without wearing gloves. In late summer 2016, 6 months before symptom onset, the farmer had cleaned the dusty sheep barn for several weeks, along with his partner and her son. The patient’s partner and her son had occasionally helped on the farm but never lived permanently on the premises. Bornavirus serology based on an indirect fluorescence antibody test and line blot with recombinant antigens [7, 14] of the patient’s partner and her son yielded negative results in February 2020.

## Discussion and conclusion

A specific etiological agent is detected in only about half of the cases with suspected infectious encephalitis [16]. The overall incidence of bornavirus-induced encephalitis in humans is unknown. However, a recent retrospective study in a diagnostic center within the endemic are in the German federal state of Bavaria has demonstrated that BoDV-1 may be responsible for a considerable proportion of fatal encephalitis cases of previously unknown origin [2]. The same may apply to endemic regions in other parts of Germany as well as in Austria, Liechtenstein and Switzerland [7, 17].

The fatal human BoDV-1 encephalitis case reported here stands out in a region previously not known for human infections. All previously published human cases had occurred > 350 km further south, in the federal state of Bavaria. The absence of a travel history and the patient’s living conditions suggest a local infection source. In contrast to all previously confirmed human BoDV-1 infections [2], the viral sequence found in this case (cluster 3) does not match the currently known animal sequences from the patient’s home region (cluster 4). However, the known cluster 3 endemic area is linked to the area of the farmer’s residence by the Elbe river valley. Further screening of shrews and diagnostic testing of animals with BD may show whether cluster 3 is additionally endemic in the northwest of Brandenburg. The geographic distribution of bicolored white-toothed shrews covers temperate regions from the Atlantic coast in the west to the Caspian Sea in the east, with its northernmost extension in Germany reaching approximately 53 to 54 degrees northern latitude [18, 19]. However, BoDV-1 appears to be endemic only in regional subpopulations occupying a rather narrow stretch from the alps to northwest Brandenburg, as indicated by virus detection in shrews and occurrence of BoDV-1-induced disease in domestic mammals and humans (Fig. 1B).

Similar to previously reported cases, the BoDV-1 transmission event remains unclear. The incubation period of human BoDV-1 infection is unknown. In naturally infected animals, it may last from a few weeks to several months [20, 21]. The patient shares potential exposure risks with other published cases, such as living in rural environments, agricultural work and animal contacts [2]. The presence of shrews on the farm could neither be confirmed nor denied, but there are reports of the reservoir species in the area [9].

This case emphasizes the need for timely information of physicians, especially neurologists, on newly identified BoDV-1 risk areas. Severe and/or fatal cases of meningoencephalitis should raise the suspicion of a bornavirus etiology. Testing of acute cases and retrospectively of fatal cases should be performed in all areas where BD is known to occur among animals. Early diagnosis is prerequisite for any therapeutic attempts.

## Data Availability

All data generated or analysed during this study are included in this published article.

## Abbreviations

BoDV-1: Borna disease virus 1
BD: Borna disease
FFPE: Formalin-fixed paraffin-embedded
RT-qPCR: Reverse transcription real time quantitative polymerase chain reaction
